# A Randomized Double-Blinded Study To Compare the Efficacy of Propofol and Thiopentone To Prevent Succinylcholine-Induced Fasciculation and Myalgia in Gabapentin-Premedicated Patients

**DOI:** 10.7759/cureus.63494

**Published:** 2024-06-30

**Authors:** Rishikesh Kumar, Vinod K Verma, Alok K Bharti, Mumtaz Hussain

**Affiliations:** 1 Anesthesiology, Indira Gandhi Institute of Medical Sciences, Patna, IND

**Keywords:** thiopentone, propofol, succinylcholine-induced myalgia, gabapentin, fasciculation

## Abstract

Background: Succinylcholine is the most used short-acting depolarizing muscle relaxant for rapid sequence induction. However, its use is associated with adverse effects, like fasciculations and myalgia. Thus, many pretreatment modalities were used to minimize or prevent these adverse effects. Our aim for this study was to compare the efficacy of propofol and thiopentone in preventing succinylcholine-induced fasciculation and myalgia in gabapentin-premedicated patients.

Methods: Eighty patients with American Society of Anesthesiologists (ASA) physical status I/II, either male or female, in the aged group of 18-60 years, and scheduled to undergo elective abdominal surgery under general anesthesia were randomly allocated into either the propofol (P) or thiopentone (T) group. Anesthesia was induced with IV fentanyl 2 µg/kg, IV succinylcholine 2 mg/kg, and either IV propofol (2 mg/kg) in group P or IV thiopentone (5 mg/kg) in group T. In both groups, oral gabapentin 600 mg was given two hours before the surgery. All patients were observed and graded for intraoperative fasciculations and myalgia during 24 postoperative hours by a blinded observer. Fasciculation grade, myalgia grade, total tramadol consumption, and demographic data were compared using a test of proportion and chi-squared test.

Results: Study results demonstrated that the use of propofol significantly decreases the severity of fasciculation at one, two, and three minutes (P < 0.001) and myalgia at two, six, and 12 hours (P < 0.001) more than thiopentone in gabapentin-premedicated patients. Tramadol consumption in both groups was insignificant (P = 0.658).

Conclusions: Propofol (2 mg/kg) is more effective than thiopentone (5 mg/kg) in decreasing the severity of fasciculation and myalgia following succinylcholine administration in gabapentin-premedicated patients.

## Introduction

Succinylcholine is still considered a drug of choice for rapid sequence induction and anticipated difficult airway management. However, its use is associated with undesirable side effects like myalgia and fasciculation, which might be distressing for patients. The incidence of fasciculation is about 95%, while the incidence of myalgia at 24 hours is approximately 50% following the use of succinylcholine [1]. Gabapentin is an antiepileptic drug that works through various mechanisms, like potentiation of gamma-aminobutyric acid transmission, reduction of glutamate-mediated excitatory transmission, and voltage-activated ion channel inhibition. The latter mechanism of action might be the reason for using these agents as a pre-emptive analgesic to prevent succinylcholine-induced myalgia [2]. Thiopentone and propofol are commonly used induction agents in anesthesia and have been shown to decrease the severity of fasciculation and, to some extent, myalgia following the use of succinylcholine. To date, numerous studies have explored drugs like diazepam, pregabalin, and rocuronium to prevent succinylcholine-induced fasciculation and myalgia. Hence, we hypothesized that the combined use of either propofol or thiopentone with gabapentin would be more effective than using either drug alone in reducing the severity of fasciculation and myalgia. None of the prior studies have compared the efficacy of propofol and thiopentone in preventing succinylcholine-induced fasciculation and myalgia severity over time in gabapentin-premedicated patients.

To fill this gap in research, we designed a study to compare the efficacy of propofol and thiopentone in preventing succinylcholine-induced fasciculation and myalgia in patients premedicated with gabapentin.

## Materials and methods

After obtaining institutional ethical committee approval from the Institutional Ethics Committee of Indira Gandhi Institute of Medical Sciences, Patna (approval 1982/IEC/IGIMS/2020) and informed written consent, 80 patients, either male or female, in the age group of 18 to 60 years, with American Society of Anesthesiologist (ASA) physical status I/II, were included in the study and scheduled to undergo elective abdominal surgery under general anesthesia. This prospective, randomized clinical trial was registered with the Clinical Trial Registry-India (CTRI/2021/03/032329), and data were recorded for research and educational purposes. The study procedures followed the guidelines of the World Medical Association and adhered to principles of the Declaration of Helsinki 2013 and good clinical practice. Patients with a history of allergy to the study drugs; chronic use of gabapentin, opioids, or sedatives; ASA grade III/IV; pregnancy; morbid obesity; known pulmonary, cardiovascular, hepatic disease, or musculoskeletal disorders were excluded.

After enrollment in the study, all patients were assessed preoperatively and were premedicated with oral ranitidine 150 mg and alprazolam 0.5 mg on the night and two hours before surgery. The patients were advised NPO as per guidelines and oral gabapentin 600 mg two hours before the surgery. Upon arrival in the operation room, a standard ASA monitor was attached, and a wide-bore intravenous (IV) cannula was secured. Randomization was done with computer-generated random numbers using sequentially numbered, opaque, sealed envelopes, allocating patients into two equal groups (n = 40 each). After preoxygenation with 100% oxygen for three minutes, anesthesia was induced with IV fentanyl 2 µg/kg, IV succinylcholine 2 mg/kg, and either IV propofol (2 mg/kg) in group P or IV thiopentone (5 mg/kg) in group T. In both groups, oral gabapentin 600 mg was given two hours before the surgery. Baseline parameters including mean blood pressure, heart rate, respiratory rate, and SpO_2_ were recorded. An independent anesthesiologist (not involved in the study) loaded the pre-calculated dose of study drugs in a syringe wrapped with black paper. Both the attending anesthesiologist and patients were blinded to group allocation. The patients were observed and graded for intraoperative fasciculations and myalgia during 24 postoperative hours by a blinded observer.

After checking ventilation, the patients were given succinylcholine to facilitate the smooth insertion of a cuffed endotracheal tube. Laryngoscopy and tracheal intubation were performed after cessation of fasciculations or one minute after succinylcholine administration. All patients were observed for the severity of fasciculations at intervals of one minute up to five minutes. The severity of fasciculation was graded as shown in Table 1.

**Table 1:** Grading of the severity of fasciculation

Grade	Description
Grade 0 (nil)	Absence of visible fasciculations
Grade 1 (mild)	Having fine fasciculations of the face, eyes, neck, or fingers without movements of the limb
Grade 2 (moderate)	Having obvious, reasonable fasciculations in more than one site body or limb movements
Grade 3 (severe)	Vigorous, sustained, and widespread fasciculations

Anesthesia was maintained on the ratio of oxygen and nitrous oxide (50%:50%) with isoflurane and intermittent vecuronium 0.01 mg/kg given every half hour until completion of surgery. Paracetamol 1 gram IV was given six hourly for postoperative analgesia. Hemodynamic monitoring was done at baseline, induction, intubation, and postoperative period. After completion of surgery, residual neuromuscular weakness was reversed with intravenous glycopyrrolate (0.01 mg/kg) and neostigmine (0.05 mg/kg), followed by smooth extubation. All patients were also observed for adverse effects at induction and intubation. The severity of myalgia was assessed after surgery at 0, two, six, 12, 18, and 24 hours in the surgical ICU. The patients were also observed for adverse effects during induction, intubation, and the postoperative period. Although efforts were made to conceal the focus on myalgia from patients (muscle pain not related to surgical pain) was graded as shown in Table 2.

**Table 2:** Grading of the severity of myalgia

Grade	Description
Grade 0 (nil)	Absence of muscle pain
Grade 1 (mild)	Minor stiffness of transient duration and localized to one site
Grade 2 (moderate)	Muscle pain to multiple sites or severe pain to one site
Grade 3 (severe)	Widespread muscle pain, severe pain to more than one site body, disability confining patient to the bed, severe muscle pain than pain of surgical site, no adequate sleep due to muscle pain

Sample size (n) calculation was based on a previous study [3] having a comparison of two proportions assuming α error of 5% and power of study of 80% and considering the incidence of fasciculation with thiopentone (78.79%) and propofol (48.48%). The sample size was 36 in each group, and adding 10% of the total sample size as a contingency, the total sample size (N) became 80 (40 in each group).

Statistical testing was used to analyze all the data using SPSS Statistics version 22.0 (IBM Corp., Armonk, NY, USA). The data were presented as mean ± SD for continuous variables and as percentage (%), ratio, and absolute number for categorical variables. Statistical tests applied for these variables were the Chi-squared test, Kruskal-Wallis test, and proportion test. P < 0.05 was considered statistically significant. Primary outcome measures were to assess the efficacy of propofol and thiopentone to prevent succinylcholine-induced fasciculation at intervals of one minute up to 5 minutes intraoperatively and myalgia during 24 postoperative hours. The secondary outcome was to measure the total amount of tramadol used as rescue analgesia to reduce myalgia during 24 postoperative hours.

## Results

**Figure 1:**
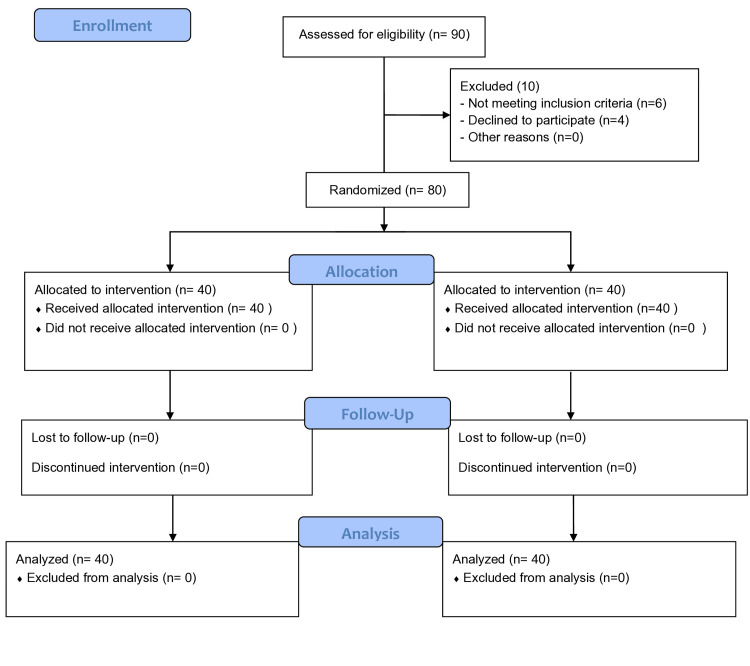
Consort flow diagram

The consort flow diagram of the studied patients is shown in Figure 1. Out of 90 patients, 80 completed the study successfully. Demographic data, including age, sex, weight, ASA class, and surgery duration, were comparable between the two groups (P > 0.05; Table 3). Hemodynamic parameters including heart rate (HR) and mean arterial pressure (MAP) between two groups at baseline, induction, and intubation were comparable (P > 0.05). Intravenous propofol was more effective than thiopentone in reducing the severity of fasciculation at one, two, and three minutes and was statistically signification (P < 0.001; Table 4), whereas no fasciculation was observed in either group at five or five minutes. Compared to intravenous thiopentone, propofol was more effective than thiopentone in reducing the severity of myalgia at two, six, and 12 hours, with statistically significant results (P < 0.005; Table 5), whereas no myalgia was reported in either group at 18 or 24 hours.

**Table 3:** Comparison of demographic data between two groups Abbreviations: ASA, American Society of Anaesthesiologists; F, female; M, male. Data are presented as mean ± standard deviation, number, or percentage.  *P < 0.05 was considered significant.Chi-squared test and t-test were used for analysis as required.

Parameter	Propofol (n = 40)	Thiopentone (n = 40)	P value	T value	*χ*^2^
Age(years) (Mean ± SD)	36.355 ± 11.74	37.77 ± 11.86	0.649	0.464	-
Weight (Kg) (Mean ± SD)	58.40 ± 11.03	59.57 ± 12.69	0.697	0.442	-
Sex (M/F)	18/22	20/20	0.654	-	0.201
ASA Grade (I/II)	28/12	31/9	0.445	-	0.267
Duration of surgery (minutes) (mean ±SD)	122.00±64.85	121.00 ± 91.92	0.958	0.056	-

**Table 4:** Comparison of the severity of fasciculation between two groups at different time intervals. Data are presented as percentages. *P < 0.05 was considered significant and **P < 0.001 was considered as highly significant.  Kruskal-Wallis test was used for analysis as required.

Time interval	Group P (n = 40) (%)				Group T (n = 40) (%)				P-value
Fasciculation grade	Nil	Mild	Moderate	Severe	Nil	Mild	Moderate	Severe	
0 min	40	0	0	0	40	0	0	0	---
	(100%)	(0%)	(0%)	(0%)	(100%)	(0%)	(0%)	(0%)	
1 min	3	17	19	1	1	6	28	5	<0.001**
	(7.5%)	(42.5%)	(47.5%)	(2.5	(2.5%)	(15%)	(70%)	(12.5%)	
				%)					
2 min	36	3	1	0	22	12	6	0	<0.001**
	(90%)	(7.5%)	(2.5%)	(0%)	(55%)	(30%)	(15%)	(0%)	
3 min	40	0	0	0	30	9	1	0	<0.001**
	(100%)	(0%)	(0%)	(0%)	(75%)	(22.5%)	(2.5%)	(0%)	
4 min	40	0	0	0	40	0	0	0	---
	(100%)	(0%)	(0%)	(0%)	(100%)	(0%)	(0%)	(0%)	
5 min	40	0	0	0	40	0	0	0	---
	(100%)	(0%)	(0%)	(0%)	(100%)	(0%)	(0%)	(0%)	

**Table 5:** Comparison of severity of myalgia between two groups at different time intervals. Data are presented as percentage. *P < 0.05 was considered as significant and ** P < 0.001 was considered as highly significant. Kruskal-Wallis test was used for analysis as required.

Time interval	Group P (n = 40) (%)				Group T (n = 40) (%)				P-value
Myalgia grade	Nil	Mild	Moderate	Severe	Nil	Mild	Moderate	Severe	
2 hours	24 (60%)	16 (40%)	0 (0%)	0 (0%)	14 (35%)	9 (22.5%)	6 (15%)	11 (27.5%)	<0.001**
6 hours	31 (77.5%)	8 (20%)	1 (2.5%)	0 (0%)	18 (45%)	11 (27.5%)	10 (25%)	1 (2.5%)	<0.001**
12 hours	35 (87.5%)	5 (12.5%)	0 (0%)	0 (0%)	23 (57.5%)	15 (37.5%)	2 (5%)	0 (0%)	0.003*
18 hours	37 (92.5%)	3 (7.5%)	0 (0%)	0 (0%)	37 (92.5%)	3 (7.5%)	0 (0%)	0 (0%)	---
24 hours	38 (95%)	2 (5%)	0 (0%)	0 (0%)	38 (95.5%)	2 (5%)	0 (0%)	0 (0%)	---

Total tramadol consumption (mg) for myalgia between two groups at 24 hours was not statistically significant. The mean ± SD for group P was 153.84 ± 51.88, and it was 165.38 ± 80.06 for group T (P = 0.658, t-value = 0.454). Two patients in group P had transient hypotension during induction. In group T, three patients had hypertension at intubation, well managed with pharmacological intervention.

## Discussion

Our study demonstrated significantly less fasciculation in the gabapentin-propofol group compared to the gabapentin-thiopentone group at one, two, and three minutes intraoperatively (P < 0.001). Similarly, postoperative myalgia was significantly lower in the gabapentin-propofol group at two, six, and 12 hours (P < 0.05). Interestingly, the maximum duration of myalgia experienced was up to 12 hours, shorter than observed in other randomized trials, possibly due to the use of oral gabapentin with either propofol or thiopentone. In our study, we also noted that the maximum incidence of fasciculation between group P and group T (92.5% versus 97.5%) was seen at one minute, while the least incidence of fasciculation between group P and group T (0 % versus 2.5%) was seen at three minutes, whereas no fasciculation was seen in either group at four or five minutes, indicating that need for a well-defined time interval between induction agent and succinylcholine administration.

Succinylcholine remains the preferred drug of choice for rapid sequence induction, and it provides excellent muscle relaxation for short surgical procedures. However, the use of succinylcholine is frequently accompanied by postoperative myalgia that might be distressing to patients [4,5].

Although myalgia is self-limiting, iatrogenic postoperative myalgia is a significant concern for anesthesiologists. Thus, various pretreatment options have been explored to decrease the severity of myalgia and fasciculation including intravenous lignocaine [6], non-steroidal anti-inflammatory drugs (NSAIDs), diazepam [7], magnesium [8], small doses of succinylcholine as self-taming [9], remifentanil [10], and gabapentin [11], but all of them come with variable success. Hence, we planned this study to compare the efficacy of propofol (2 mg/kg) and thiopentone (5 mg/kg) to prevent succinylcholine fasciculation and myalgia in gabapentin-premedicated patients.

We compared our results with Parmar et al.'s study [3], which investigated the usefulness of propofol to thiopentone to prevent succinylcholine-induced fasciculations and myalgia. They took 99 patients in their study and randomly allocated them into three equal groups (propofol group 1, propofol group 2, and thiopentone group), and succinylcholine was used in a dose of 2 mg/kg to facilitate laryngoscopy and intubation. Finally, they found lower incidence of both fasciculation and myalgia in propofol groups as compared to the thiopentone group (P < 0.001). Our findings align with theirs, suggesting gabapentin-propofol is superior to gabapentin-thiopentone in this regard.

Another similar study by Hika et al. [12], who compared the efficacy of thiopentone and propofol thiopentone sodium in preventing succinylcholine-induced myalgia and fasciculation in elective surgical patients. They took 80 patients in their study and were randomly divided into two equal groups - propofol group (3.5 mg/kg) and thiopentone group (5 mg/kg) and succinylcholine was given in the dose of 2mg/kg to facilitate intubation, results demonstrated significant reduction in incidence and severity of fasciculation and myalgia between group P and group T. They concluded that propofol was superior than thiopentone in reducing succinylcholine-induced fasciculation and myalgia.

On comparing the results of this study to Hika et al.'s study, we found that the severity of fasciculation and myalgia was statistically more significant (P < 0.001). The greater statistical significance of our study compared to Hika et al. might be attributed to the role of either gabapentin-propofol synergism or a combination of gabapentin-thiopentone.

Interestingly, the duration of myalgia in our study was up to 12 hours, contrary to previously published studies, where the duration of myalgia was in the range of 24-48 hours. Again, the most likely explanation would be the synergistic action of gabapentin with either propofol or thiopentone. The most likely mechanism of gabapentin to decrease succinylcholine-induced myalgia postoperatively is the binding of gabapentin to α2‑subunit of voltage-dependent calcium channels [13], thus reducing calcium influx into nerve terminals and subsequently inhibiting the release of excitatory neurotransmitters like aspartate and glutamate; as a result, postsynaptic excitability [14] and finally myalgia pain are reduced.

Differences in the HR and MAP between the two groups were not significant, which might be the result of the use of an optimal dose of induction agents. The total tramadol consumed as rescue analgesia in the postoperative period for myalgia was statistically insignificant (P = 0.658) because of the oral use of gabapentin in both groups. There were no adverse side effects like bradycardia, arrhythmias, and bronchospasm seen between the two groups.

Our study limitation was that we could not measure fasciculation and myalgia objectively and all the patients included in the study had ASA physical status I and II; as such, caution must be exerted while generalizing the results to ASA physical status III and IV patients.

## Conclusions

Propofol 2 mg/kg IV is more effective than thiopentone 5 mg/kg IV in reducing the severity of succinylcholine-induced fasciculation and myalgia in patients premedicated with gabapentin. Stable hemodynamics was observed with the use of these drugs, and it does so without any significant adverse effects. Total tramadol used as rescue analgesia for myalgia during the first 24 hours postoperatively was comparable between the thiopentone and propofol groups following succinylcholine administration.
